# Demystifying programme theories of co-production in health and welfare: An interview study on new researchers’ systems perspectives

**DOI:** 10.1186/s12961-025-01368-y

**Published:** 2025-07-10

**Authors:** Annika Nordin, Sofia Kjellström, Ann-Christine Andersson

**Affiliations:** 1 Department of Quality Improvement and Leadership, Jönköping Academy for Improvement of Health and Welfare, School of Health and Welfare, Jönköping University, Jönköping, Sweden; 2 The Child Health Care Service, Region Jönköping County, Jönköping, Sweden

## Abstract

**Background:**

Coproduction is an inclusive approach for improving health and social care services, and coproduction research mostly focuses on participating stakeholders who are not researchers. Programme theories are important for designing, evaluating and disseminating change initiatives; however, few empirical studies on quality improvement initiatives or coproduction projects include explicit programme theories. This study addresses these knowledge gaps by describing new researchers’ initial implicit programme theories of coproduction from three different system perspectives.

**Methods:**

This is a cross-sectional interview substudy that includes 12 respondents. The respondents are new researchers (doctoral students) in Samskapa, an international research programme. The respondents conduct their studies in their own national contexts: Western Europe and North America. The interviews focus on their thoughts and experiences of coproduction, and the data are analysed using directed content analysis based on central concepts of programme theory. Coded statements are additionally coded for the system perspective they refer to. To describe programme theories of coproduction from micro-, meso- and macrosystem perspectives, a latent interpretation of the data is carried out. The consolidated criteria for reporting qualitative research (COREQ) checklist for qualitative studies was used to assure quality standards.

**Results:**

A generic programme theory of coproduction is suggested: If microsystem actors collaborate, facilitated by mesosystem mediators and supported by macrosystem managements’ feedback and engagement, then coproduction will occur and health and welfare systems will improve.

**Conclusions:**

Coproduction projects are complex interventions that exhibit equifinality – a principle from open systems theory which posits that similar outcomes can be achieved through multiple, distinct pathways. Programme theories of coproduction from several system perspectives can be merged into a generic programme theory, which in turn can capture the interventions’ complexity.

**Supplementary Information:**

The online version contains supplementary material available at 10.1186/s12961-025-01368-y.

## Background

In quality improvement work, the use of programme theories has proven to be valuable for successful design, execution and evaluation [1–3]. The value of programme theories has recently been further emphasized by the updated Medical Research Council framework, which highlights programme theory as a core element for developing and evaluating complex interventions [4].

Originating from evaluation research [5], programme theories provide an overview of the logical relationships between the central concepts of actors, the programmes’ activities, expected outcomes and the assumptions on how the activities lead to the outcomes [2, 6]. To clarify how the desired outcomes are presumed to be achieved, a verbalized programme theory needs to include explanations of the improvement initiative’s “if”, “then” and “so-what” ([2], p. 232). In a study on children with intellectual disability receiving residential support in Sweden, a programme theory was used to evaluate an improvement initiative including coproduction approaches [7], and in a study on an intervention for people with haemophilia, a programme theory was cocreated [8]. Even though a generic programme theory template for coproduction has been suggested [9], few empirical studies on quality improvement or coproduction include explicit programme theories. This absence hampers external parties’ ability to evaluate whether and how improvement initiatives are relevant in their own context. The absence of explicit programme theories also makes the identification of necessary local adjustments for a successful replication of improvement initiatives more difficult. Consequently, the absence of explicit programme theories complicates the spread of successful improvement initiatives [2].

Coproduction has been described as a new quality improvement paradigm in healthcare ([10], p. 3). It is an inclusive approach for improving service quality in health and social care [10–12]. There are various definitions [13] and meanings of coproduction [14], and this article adopts the definition suggested by Osborn et al.: “the voluntary or involuntary involvement of public service users in any of the design, management, delivery and/or evaluation of public services” ([15], p. 640). This broad definition is well-suited for this article, which reports on an empirical study of initial implicit programme theories of coproduction among a group of new researchers – doctoral students – studying coproduction in both health and social care services at several system levels in Swedish and English-speaking contexts.

The literature on coproduction encompasses a range of models, including conceptual/theoretical, practice-oriented, and typological frameworks. Practice-oriented models – especially those originating in health services research and quality improvement – have often not been grounded in conceptual traditions from disciplines such as public administration, management or sociology [16]. A recent literature review further identifies six mechanisms – intentions, assets, dialogue, documentation, interpretation, and understanding – are foundational elements that underpin various models of coproduction [17].

Although coproduction has been highlighted as a promising research approach, it has also been described as placing great demands on researchers. This article does not aim to weigh researchers’ possible “additional costs” for doing coproduction research [18] against the egalitarian values and relevant outputs that coproduction is proposed to result in [19]. Instead, our interest lies in the new researchers’ own thoughts and experiences of coproduction. In coproduction research, the focus is mostly on the participating stakeholders who are not researchers. These stakeholders, such as professionals and service users, are often described in terms of gender, age and experience, but participating researchers are rarely described in the same detail [20]. Ignoring researchers as stakeholders in coproduction entails a missed opportunity to develop deeper knowledge about their theoretical and empirical understanding of coproduction.

Clinical microsystem theory has proven helpful in driving quality improvement in healthcare by emphasizing the importance of addressing deficiencies at several system levels simultaneously [21]. The theory centres on three system levels, which can also be understood from three different system perspectives. The clinical microsystem represents the synchronous place and time where health and social care services are delivered and includes the service users using the specific service. The clinical microsystems are embedded at the mesosystem level (organizational support structures), which in turn are embedded at the macrosystem level (the broader health system). The value of using the micro, meso and macro perspectives is also suggested in the generic programme theory template of coproduction by Brix and colleagues [9].

This study aims to demystify coproduction programme theories by exploring new researchers’ initial programme theories of coproduction. The research questions are as follows:

- How can initial implicit programme theories of coproduction at several system levels be described?
- How can the initial implicit programme theories of coproduction be interpreted?

## Methods

### Study design and researchers

This study is a substudy in the research programme Samskapa, whose published study protocol states that the purpose relates to the measurements, mechanisms, management, and models of coproduction. These study areas are in the Samskapa research programme called the 4 M’s of coproduction [22].

The substudy is a cross-sectional interview study using directed content analysis [23]. The consolidated criteria for reporting qualitative research (COREQ) checklist for qualitative studies was used to assure quality standards [24]. The interviewers (A.N. and S.S.) were female PhDs with extensive experience in qualitative research. One interviewer is British, and one is Swedish, with extensive knowledge of English. All the authors were experienced in the research field of quality improvement and leadership and have complied with the International Committee of Medical Journal Editors (ICMJE) guidelines for authorship.

### Interview guide and data collection

To test the interview guide five pilot interviews were conducted (S.K.) with doctoral students in the Samskapa research programme who volunteered to participate. The interview guide included questions on the definition of coproduction, the respective research projects, and the four Ms. Subsequent discussions among the Samskapa researchers revealed the need to incorporate more reflective questions and follow-up questions into the semistructured interview guide. These additions aimed to clarify time-related aspects of activities and experiences. The data from the pilot interviews were not included in the data analysis.

Since the respondents and the researchers were part of the same research programme, an in-depth ethical analysis was conducted, resulting in an ethics application approved by the Ethics Review Authority in Sweden (2019-06372). One interviewer (A.N.) was the co-supervisor of two of the doctoral students. They were therefore offered the opportunity to be interviewed by an external researcher, which was declined.

The developed interview guide included five topics (the research project, the four Ms, challenges, experiences of being a change agent and a researcher, and learning and thinking of coproduction). All doctoral students in the Samskapa research programme were informed about the study purpose and process in an invitational letter. It emphasized that participation was voluntary and described the study focus as being on the doctoral students’ thoughts and experiences of coproduction, and not on their academic achievements. In all, 12 (of 15) doctoral students agreed to participate. These 12 respondents signed an informed consent form. Six respondents spoke mainly Swedish, and six respondents spoke mainly English. The respondents conducted their studies in their own national contexts: Western Europe and North America.

To protect their integrity, the respondents and their research projects are not described in detail here. Their study contexts were clinical care, elder care, habilitation, mental healthcare, organizational development, and rehabilitation. All the respondents studied projects that, to varying degrees, included coproduced approaches, and they all had professional work experience from the empirical context of their studies. Five respondents studied projects using a structured experience based co-design (EBCD) approach [25], while the others studied locally developed approaches that emerged over time. Examples of such approaches were series of workshops with staff and service users to improve the care experience or service quality. Other examples were joint developments of tools and materials aimed at strengthening the voice of patients and service users. In all but two cases, the studies were in an initial stage at the time of the interview.

Eleven interviews were conducted online, via Zoom or Teams, and one interview was conducted at the doctoral student’s workplace. The interviews were conducted in English, lasted approximately 1 h and were recorded and transcribed (178 pages). Notes were taken during the interviews to support follow-up questions but were not part of the data analysis. All quotes have been edited for grammatical accuracy, and respondents have provided their consent for use. To ensure their privacy, respondents are identified in the manuscript by numbers rather than names.

### Coding and data analysis

The codes were developed deductively on the basis of the three central concepts of programme theory: actors, activities, and expected outcomes and measurements [2, 6]. The three authors discussed the definitions of the codes until a consensus was reached (Table 1).

**Table 1:** Definitions of codes

Code	Definition
Actors	All those who actively participated in the coproduction initiatives – individuals, groups or organizations. Actors could, for example, be service users, patients, clients, customers, their relatives or professionals. Actors could have different roles in different contexts.
Activities	All deliberate actions or activities carried out by any of the actors in the coproduction initiatives.
Outcomes and measurements	The expressed arguments for, and expectations of, the coproduction initiative and all statements regarding quantitative or qualitative measurements.

The data analysis consisted of five steps. In the first step, the first author coded statements related to the three central concepts, numbered them sequentially and recorded them in an Excel file. In the second step, the authors A.N. and A.C.A. individually coded the statements for the system perspective they referred to (micro, meso or macro). In the third step, the authors compared their coding and engaged in a dialogue until consensus was reached. During this process, the initial coding was also double-checked and re-coded when perceived as necessary. After the finalized coding A.N. and A.C.A. had two meetings to analyse the results. In this fourth step, A.N. and A.C.A. made a latent interpretation of the results to describe their underlying meaning in terms of implicit programme theories from micro, meso and macro perspectives in a joint workshop. In the workshop, the codes for each perspective were projected on a large screen, and A.N. and A.C.A. had a reflective conversation to jointly formulate the underlying implicit programme theories. These preliminary programme theories were tested against the results and rewritten a number of times. The final interpreted programme theories were included at the end of each result subsection below. Finally, they were merged into a generic initial programme theory of coproduction, which was included at the end of the results section. S.K. was involved in the process of analysis throughout all steps and contributed reflections, questions and a principal review of the results. To avoid reducing member checking to a restricted acceptance of a text, the respondents were not only sent a copy of the preliminary manuscript but also invited to one voluntary dialogue meeting as a fifth step of the analysis. Four respondents chose to participate. They discussed the manuscript in pairs, taking notes. Subsequently, their reflections were shared with the researchers, who documented the conversation.

## Results

### Microsystem perspective

#### Actors

All the respondents mentioned professionals and users, and they served as a core set of actors at the microsystem level. Users were described as a multifaceted category, such as patients, clients, customers or people with different diagnoses or care needs. Actors were generally described as being included on the basis of their knowledge and experience and not on the basis of their organizational representation. However, in cases of large and complex interventions, the need to include actors from several organizations was recognized, using the examples of care organizations and municipalities. Most respondents included family members and/or next of kin and some felt this category of actors could be involved more widely. Beyond the core set of actors, the composition of actors was believed to evolve over time. One respondent expressed this by saying, “My plan is not to decide the stakeholders from the start, but I absolutely have an idea” (Respondent 2).

#### Actions

The respondents mainly talked about the overall interventions, without specifying the included activities. They spoke of “changes”, “interventions”, “codesign groups” and “prototypes of interventions” without describing the activities involved. When activities were mentioned, they were described in general terms, with some exceptions. Various forms of structured and facilitated meeting places have been developed. One example was “learning cafes”, which are a type of group learning session [26]. In the learning cafes, patient groups and healthcare staff met to discuss both self-care and professionally provided care. One respondent described how the cafes worked; “one patient or client…tells the story how it was when he or she got sick…and when you listened to such a story…we discussed around the table if this was the best we could do” (Respondent 1). Another example was codesign meetings in which a variety of actors collaboratively conducted improvement work. A further example of a structured activity was the use of pre-agreed-upon cooperation contract. To develop a thorough understanding of patients’ experiences with care, patient stories were essential. Here, one or more patients were accompanied throughout the whole care process, crossing organizational and professional boundaries.

Different types of informal meetings to test and work with improvements in cooperation were mentioned. One respondent specifically described the meetings as the place where improvement tools and models were explored and applied together with the actors. The informal meetings were recognized by several as being used to consult and discuss with different actors. Others stated that the dialogue should not only take place at meetings but also be continuous. They argued that the dialogue with users and professionals supported the continuous management of the work. The respondents who carried out experienced-based codesign projects [25] rather unreflectively described the steps of the model as activities to tic-off (that is, setting up, engaging staff and gathering experience, engaging patients and gathering experience, codesign meetings, small codesign teams and a celebration event). However, they also described their personal experiences and challenges with such projects in a reflective way.

The respondents talked about the activities as both something they did solitarily (“I have done”) and something they did, together with others (“we have done”). The empirical interventions were more consistently described as joint activities, and the research was described as individual activities.

#### Outcomes and measurements

Several respondents could not provide examples of specific predefined or expected outcomes. They explained that it was important to formulate expectations of outcomes together with all the actors involved, which had not yet been done. The respondents assumed that expectations of outcomes would be developed during the project. Some concrete examples concerned the development of effective patient processes and tools that relatives and professionals could use. In addition, outcomes have been described more theoretically. The respondents described how patient collaboration could bring a patient-centred rigour to the projects and serve as a constant reminder of the project’s objectives. In this way, coproduction was seen as a long-term resource-efficient approach to continuously validate projects. Coproduction was also described as enhancing understanding, trust and respect, which could impact the future handling of problems. The inherent democratic value of coproduction was also emphasized.

There were few specified measurements at the start of the projects. The respondents felt that it was important but difficult to measure and believed that measurements would be developed collaboratively at a later stage. In one project, measures that had been used in other projects were initially used. It proved to be inadequate in the current context and its use was discontinued; “I realized that those measures were just too broad and just…stupid” (Respondent 8). The need for qualitative measurements to gain a deeper understanding of users’ experiences was mentioned once.

#### Programme theory of coproduction from a microsystem perspective

If several different types of actors collaborate in activities within coproduction projects, then user value will be created and measurements will develop, even when the coproduction projects do not have clearly predefined objectives.

### Mesosystem perspective

#### Actors

The mesosystem level contained several different types of actors not mentioned at the microsystem level, such as managers and management teams. Their role was to support the projects and motivate coproduction activities in the organization. Other important actors were human resources (HR) staff, trainers and various administrators. Larger projects needed actors and mandates from different organizations, such as hospital care, primary care and municipalities. The respondents also talked about mediators, which they described as important roles that actors could adopt in different situations. The role of the mediator was to facilitate coproduction and increase understanding at the microsystem level. People who acted as mediators had a health or social care profession or were persons with lived experiences. They had different titles, such as coach, facilitator, improvement manager, living library representative or mentor. One respondent described the different roles on the meso level, also including the own role as researcher: “There is a project manager who is running that project, and I’m leading kind of from the back and giving advice on experience codesign and how that works. And then there is a facilitator that will specifically be running the sessions” (Respondent 10).

#### Actions

There were few descriptions of actions at the mesosystem level. The few we found were not different from the actions at the microsystem level and therefore were hard to differentiate. Examples were meetings aimed at improving the process but not a single microsystem level issue. Such meetings could contain subjects such as coordinating coproduction activities or making plans for distribution between different microsystems’ ideas.

#### Outcomes and measures

Defining outcomes, or even expected or possible outcomes at the mesosystem level, was difficult. The few examples given were about organizational innovation (large change) to accomplish better access to health and social care services, to develop a sort of toolbox to support patients and their next-of-kin to be able to coproduce more actively at the microsystem level, and to increase patients’ feelings of security. Several respondents mentioned learning and learning activities as an expected outcome. One respondent mentioned power as a valuable outcome: “We could benefit from giving individuals more power or information to act upon and be cocreators, whether it is the whole healthcare system or their own care” (Respondent 9). The learning activities included, for example, measurements and coproduction activities, or how to involve external actors (patients and/or next of kin) earlier. Moreover, the measurements were sparse, and most respondents were not even able to answer the questions. One said that no measurements had been developed yet but that they hopefully would develop over time. Another respondent referred to measurements developed in a previous study. These measurements were now used in the project as a basis for measuring patient-reported outcomes, number of meetings and number of participants. The possibility of using IT systems to support coproduction was exemplified. There were also concerns about how to inform and teach others about how patients experienced care, especially administrators. Overall, measurements at the mesosystem level were found to be difficult and not established at the initial stages of the doctoral students’ studies.

#### Programme theory of coproduction from a mesosystem perspective

If mediators facilitate meetings for a wide variety of actors from different organizations in coproduction projects, then legitimacy and learning will occur and outcomes at the microsystem level will improve.

### Macrosystem perspective

#### Actors

Actors at the macrosystem level were described mainly as representatives from different organizations, such as stakeholders, who were affected in some way. Organizational cooperation between national expert groups or diagnosis organizations, such as experts or advisory boards, was an example.

Persons with lived experiences were mentioned as actors, although they were not included in the projects. The need for mediators, including steering board members and national programme managers, was described at the macro level. One respondent described this as complex: “So, there are many stakeholders, at different levels, who play a role in this” (Respondent 3). Researchers also appeared at this level but their role could be complex, as they for example could be both actors and project leaders in the form of an action research approach.

#### Actions

Few respondents talked about actions at the macrosystem level. The brief examples included high-level management and political influences, or some larger projects, such as national incentives or national education for coproduction mediators. A national collaboration to improve the lives of people with different conditions and support their needs was also mentioned.

#### Outcomes and measurements

The respondents stated that their motivation to engage in coproduction projects mainly stemmed from their own disappointment from their experiences with health and social care services at the macrosystem level. Some stated that they wanted to improve the ability of health and social services to adopt a holistic view and put the user in the centre. Measurements from a macrosystem-level perspective were limited. All of the few examples mentioned were about feedback being important at all system levels. The respondents wanted to make a difference and contribute to creating better health and welfare systems. By one respondent this was described as “It was a big vision, it wasn’t a little ‘can we coproduce our own care’, it was like we want to have a very different relationship” (Respondent 4).

#### Programme theory of coproduction from a macrosystem perspective

If the management of organizations affected by coproduction projects supports feedback and engagement at the micro and mesosystem levels, then better health and welfare systems will be created.

#### Generic initial programme theory of coproduction

If microsystem actors collaborate, facilitated by mesosystem mediators and supported by macrosystem management feedback and engagement, then coproduction will occur and health and welfare systems will improve.

## Discussion

This study demystifies programme theories of coproduction in several ways. It reveals that the new researchers (the doctoral students participating in the study) had implicit programme theories of coproduction from several system perspectives at the outset of their research projects. The study also shows that these programme theories need to be refined and improved to accommodate to the complexity inherent in coproduction projects.

### Implicit programme theories from several system perspectives

A programme theory is a core element in a complex intervention and according to the Medical Research Council framework, the programme theory needs to be refined and re-tested to provide a deeper understanding of the intervention [4]. Our study contributes to the framework by illustrating that a complex intervention can be underpinned by programme theories from several perspectives, all of which need to be refined and retested. The new researchers with previous experience working with coproduction involved a greater number of actors at the microsystem level. This suggests that previous projects helped them gain a clearer understanding of critical actors and their significance, which is an important practice in coproduction leadership [27]. By analysing the new researchers’ programme theories, a generic programme theory for coproduction is suggested. This generic programme theory may untangle some of the complexity of coproduction projects.

### Development of programme theories (and measurements) along the way

The model for improvement developed by the Associates in Process Improvement [28] has been fundamental to improvement work. The model emphasizes the need for clearly defined project objectives, and it has even been argued that “the more specific the aim is, the more likely improvement is to occur” ([29], p. 930). This contradicts the new researchers’ statements, as they did not highlight clearly defined objectives at the start of their coproduction projects. Instead, they described the objectives as generally positive values that could be clarified later, in collaboration with involved actors.

The new researchers expected their projects to develop in these value directions – as in a particular development trajectory – which corresponds to how changes in complex systems are described [30]. The idea that their programme theories of coproduction preferably could be clarified during the course of the projects also applied to measurements, which are also emphasized in the model for improvement [28]. None of the new researchers claimed to have a ready-made set of measurements at their project launches. Although they described some frustration with the difficulty of measuring coproduction, they noted that measurements were a challenge to address later, in collaboration with involved actors.

### Equifinality of coproduction projects

“Equifinality” was originally coined by von Bertalanffy and is a principle of open systems theory that describes how complex systems can achieve similar outcomes through different pathways [31, 32]. This means that individuals or teams may take different actions but still produce the same results. Equifinality thus clarifies that there are multiple paths to success, supporting adaptability and creative problem-solving. The programme theories from micro, meso-, and macrosystem perspectives in this study all contain an openness to the possibility of coproduction projects taking different routes, which can be an indication of the new researchers being grounded in an understanding of complex systems theory and equifinality. A related interpretation is that the new researchers were unsure of what actions needed to be taken but that they were confident that having a ready-made plan was not crucial, as coproduction inevitably requires continuous adaptability.

### Implications

Previous research has emphasized the importance of programme theories to evaluate and better understand improvement initiatives [1, 2] or complex interventions [4]. This is particularly important in coproduction, which involves complex and social phenomena [9]. While theory is essential for understanding and explaining phenomena, excessive abstraction or overly idealized models can make research appear disconnected from practical, real-world contexts [33]. In contrast, the programme theories revealed in this study are concise and accessible, which suggests they may be particularly useful for practitioners. These theories can serve as guiding principles when developing local programme theories and act as reminders that such theories must incorporate multiple system perspectives. In particular, the generic programme theory can help communicate and clarify the foundational principles underlying the design of coproduction projects. The openness of the programme theories suggests that coproduction is not a process that simply occurs or is implemented. Instead, coproduction can be understood as a seed that requires the right conditions to grow. At the micro level, these conditions involve social interactions; at the meso level, they involve the facilitation of these interactions; and at the macro level, they involve leadership support and broader institutional endorsement of coproduction efforts. Framing coproduction as a social, dynamic and complex phenomenon underscores the inadequacy of traditional project planning and management approaches. The description of the logic that characterizes coproduction can also be valuable for applications for funding and research clearance.

With the equifinality of coproduction revealed, the need for models supporting equifinal practices becomes clear. One such example is the model for balanced measurement of coproduction [20]. Instead of identifying specific measurements of coproduction, the model emphasizes the importance of measuring three perspectives of coproduction (outputs, outcomes and experiences of participating in coproduction). In this way, the model can guide coproduction actors to jointly identify relevant measurements, without imposing a specific measurement.

When a new area of knowledge is entered, most people are at a novice stage. The Dreyfus model describes progress during a learning journey towards becoming an expert [34]. With respect to a new field of research, the new researchers in this study had few rules or instructions to learn from, and their skill development was thus probably more implicit.

### Future research

Our study focuses on new researchers’ programme theories of coproduction from different systems perspectives. A suggested next research step is to study how these programme theories evolve as researchers gain more experience. Our study serves as a baseline for a qualitative longitudinal study on the sense-making of coproduction, and in an ongoing study we address the need to learn about the emergent understanding of coproduction and new researchers’ implicit competence development over time.

### Strengths and limitations

In qualitative studies, the power of information depends on the purpose of the study, the specificity of the sample, the use of established theory and the quality of dialogue [35]. The strategic choice of respondents is also particularly important in qualitative studies [36]. In our study, we pursued high information power through a narrow study purpose with a clear focus on the specific experiences of the new researchers. Furthermore, we conducted lengthy interviews with open-ended questions based on the interview guide. The open-ended interview questions and the lengthy interviews (exceeding 1 h) in this study provided rich data and information power, which are more central aspects of validity than saturation in qualitative research [37].

In qualitative research, member-checking has been promoted to safeguard credibility and trustworthiness [38]. Nonetheless, there is no clear evidence that member-checking contributes to research quality, especially when the study aims to develop a generalized theory [39]. Against this background, the purpose of the dialogue meeting was to present the results and jointly explore the programme theories and their implications. At the dialogue meeting it became apparent that the notions of micro, meso and macro could also be understood as different system perspectives; consequently, this has been clarified in the manuscript. In the dialogue meeting, Swedish respondents claimed that the English language was not perceived as a constraint in the interviews but that this was difficult to evaluate afterwards. Other results from the dialogue meeting was that the word “then” was inserted after the comma in all program theories to make them more consistent with how programme theories are suggested to be formulated [2]. The respondents also noted that their present interpretations of the programme theories may be influenced by the time that has passed since the interview. This may be true, and the emergent aspect of the understanding of coproduction will be addressed in our qualitative longitudinal study.

## Conclusions

Recognizing coproduction as a social, dynamic and complex process is essential for the fair and accurate planning, implementation and evaluation of coproduction projects. Making programme theories explicit from several system-level perspectives can facilitate the understanding of coproduction in complex systems. The continuous evolution and refinement of an intervention’s programme theory can, as Skivington et al. put it, promote complex interventions that provide “solutions to the real-world practice” ([4], p. 2).

## Supplementary Information

Additional file 1.

## Data Availability

No datasets were generated or analysed during the current study.
